# A metagenome-level analysis of a microbial community fermenting ultra-filtered milk permeate

**DOI:** 10.3389/fbioe.2023.1173656

**Published:** 2023-05-17

**Authors:** Kevin A. Walters, Geethaanjali Mohan, Kevin S. Myers, Abel T. Ingle, Timothy J. Donohue, Daniel R. Noguera

**Affiliations:** ^1^ Department of Bacteriology, University of Wisconsin-Madison, Madison, WI, United States; ^2^ Wisconsin Energy Institute, University of Wisconsin-Madison, Madison, WI, United States; ^3^ Great Lakes Bioenergy Research Center, University of Wisconsin-Madison, Madison, WI, United States; ^4^ Department of Civil and Environmental Engineering, University of Wisconsin-Madison, Madison, WI, United States

**Keywords:** microbiome, dairy coproduct, fermentation, lactic, succinic, butyric, hexanoic, chain elongation

## Abstract

Fermentative microbial communities have the potential to serve as biocatalysts for the conversion of low-value dairy coproducts into renewable chemicals, contributing to a more sustainable global economy. To develop predictive tools for the design and operation of industrially relevant strategies that utilize fermentative microbial communities, there is a need to determine the genomic features of community members that are characteristic to the accumulation of different products. To address this knowledge gap, we performed a 282-day bioreactor experiment with a microbial community that was fed ultra-filtered milk permeate, a low-value coproduct from the dairy industry. The bioreactor was inoculated with a microbial community from an acid-phase digester. A metagenomic analysis was used to assess microbial community dynamics, construct metagenome-assembled genomes (MAGs), and evaluate the potential for lactose utilization and fermentation product synthesis of community members represented by the assembled MAGs. This analysis led us to propose that, in this reactor, members of the *Actinobacteriota* phylum are important in the degradation of lactose, via the Leloir pathway and the bifid shunt, and the production of acetic, lactic, and succinic acids. In addition, members of the *Firmicutes* phylum contribute to the chain-elongation-mediated production of butyric, hexanoic, and octanoic acids, with different microbes using either lactose, ethanol, or lactic acid as the growth substrate. We conclude that genes encoding carbohydrate utilization pathways, and genes encoding lactic acid transport into the cell, electron confurcating lactate dehydrogenase, and its associated electron transfer flavoproteins, are genomic features whose presence in *Firmicutes* needs to be established to infer the growth substrate used for chain elongation.

## 1 Introduction

The microbial conversion of organic industrial residues into valuable fermentation products is an attractive approach to both increase the value of these feedstocks and contribute to a decarbonized bioeconomy (Han et al., 2019; Asunis et al., 2020; Wadler et al., 2022). Anaerobic microbial communities have long been used in the conversion of complex organic waste streams into biogas (i.e., anaerobic digestion) (Obileke et al., 2021), and they also play a role in the upgrading of feedstocks into higher-value fermentation products (Scarborough et al., 2022a). An array of valuable fermentation products can be generated by natural microbial communities, but there are knowledge gaps on how to harness these communities to optimize the production of a specific targeted end-product, with fermenting communities oftentimes simultaneously accumulating several fermentation products (Liu et al., 2020; Candry and Ganigué, 2021). We are interested in developing predictive tools for optimization and control of fermentation pathways and hypothesize that features could be identified within the genomes of abundant community members to allow for the inference of their metabolic functional roles. Here we elucidate genomic features and metabolic pathways of a microbial community fermenting a lactose-rich residue into potentially valuable products.

Medium chain fatty acids (MCFAs, monocarboxylic acids of carbon chain length 6–8), lactic acid, succinic acid, and butyric acid are high-value organic acids commercially produced by abiotic and biotic approaches that can be generated by fermentation of carbohydrate-rich materials (Dwidar et al., 2012; Spirito et al., 2014; Biddy et al., 2016). These organic acids could serve as target end-products of mixed-culture fermentation technologies that use organic wastes as feedstocks provided that approaches are developed to efficiently metabolize the feedstock while maximizing the accumulation of a targeted product. Mixtures of these organic acids are produced during mixed-culture fermentation of carbohydrate-rich residues from the dairy and other industries (Duber et al., 2018; Han et al., 2019; Fortney et al., 2021). From the dairy industry, ultra-filtered milk permeate (UFMP) stands out as a large-volume (American Dairy Products Institution, 2019) and low-value coproduct that could be used as feedstock for fermentation (Talabardon et al., 2000; Wang et al., 2009; Tarapata et al., 2022).

A diverse array of metabolic pathways may be involved in fermentation of UFMP. Lactose, the main organic component of UFMP (Menchik et al., 2019), can be metabolized via the tagatose-6-phosphate pathway (Bissett and Anderson, 1973) or the Leloir pathway (Maxwell et al., 1962). Lactic acid can be produced via Embden-Meyerhof-Parnas (EMP) glycolysis (homofermentation), along with acetic acid via the bifid shunt (Pokusaeva et al., 2011), or with ethanol and carbon dioxide via the phosphoketolase pathway (DeMoss et al., 1951). Succinic acid can be produced from central metabolism via four known metabolic routes (Song and Lee, 2006; Cao et al., 2013). Butyric, hexanoic, and octanoic acids are often produced through chain elongation via the reverse β-oxidation pathway (Buckel and Thauer, 2013). With the wide array of metabolic pathways available to lactose-fermenting communities, elucidating specific features of these metabolic pathways that could be used to functionally classify community members may enhance our ability to predict microbial function and identify approaches to engineer microbiomes for generation of specific fermentation products (Myers et al., 2023).

We explore here the range of fermentation products that could be made from UFMP, analyze the abundant members of the fermenting microbiome, evaluate the genomic content of community members, and use these data to propose the potential role of different community members in the production of different fermentation products. To accomplish our goals, we operated a bioreactor under similar conditions to those used to obtain MCFA-producing microbial communities using other feedstocks (Scarborough et al., 2018b) and observed the microbial community dynamics that lead to different profiles of fermentation product accumulation over time. Knowledge gained in this study helps expand metagenomic-based understanding of lactose-upgrading microbial communities and offers new insights toward developing models and strategies that can optimize mixed-culture microbial fermentations.

## 2 Materials and methods

### 2.1 Materials

UFMP was supplied by the Center for Dairy Research (Madison, WI, USA) and stored frozen at −20°C until use. The microbial inoculant was collected from an acid-phase anaerobic digester at the Madison Metropolitan Sewerage District (Madison, WI, USA) and used right after collection for bioreactor inoculation.

### 2.2 Bioreactor system

A continuous stirred tank reactor with a 3-L working volume was constructed in-house using a 4-L glass Erlenmeyer flask and a butyl rubber stopper. This bioreactor was continuously stirred at 200 rpm and heated to 35°C with a stir plate and heat tape that was temperature controlled using a thermocouple/controller system. The pH was monitored and maintained at 5.5 with a pH probe, a pH controller, and automated addition of 5M NaOH, when needed. The reactor was seeded with equal volumes of feedstock and inoculum from an acid-phase digester operated in the Madison Metropolitan Sewerage District’s wastewater treatment plant (Madison, WI). The feedstock, UFMP amended with 400 mg N/L (as NH_4_Cl), was continuously fed at a rate of 0.5 L/day throughout 282 days of operation resulting in solids and hydraulic retention times of 6 days. Temperature and pH were maintained constant at 35°C and 5.5, respectively. Care was taken to minimize input of air into the vessel.

### 2.3 Sample collection and storage

Samples were collected roughly every 6 days from the bioreactor for chemical analyses and DNA extraction. Fully mixed reactor broth was collected, some of which was stored at 4°C, and the rest centrifuged at 10,000 *g* for 10 min. The supernatants of centrifugated samples were passed through a 0.22-µm polyethersulfone membrane syringe filter and stored at −20°C or 4°C. The microbial biomass pellets remaining after centrifugation were stored at −80°C.

### 2.4 Analytical tests

Analytical tests were performed on UFMP samples and on samples collected from the bioreactor. Manufacturer’s protocols were used in each case. Chemical oxygen demand (COD) was measured on filtered and unfiltered samples using COD2 mercury-free high range (20–1,500 mg/L) digestion vials (2565115, Hach, Loveland, CO, United States). Total nitrogen was measured on filtered samples using a high range total nitrogen reagent set (TNT, 2714100, Hach). Orthophosphate assays were performed on filtered samples using PhosVer 3 phosphate reagent powder pillows (216069, HACH). Biomass was estimated by quantifying the volatile suspended solids using Method 2540G (American Water Works Association and Water Environment Federation, 2005).

The concentrations of carbohydrates (lactose, glucose, galactose), some organic acids (formic, acetic, lactic, and succinic) within filtered samples were quantified by high-performance liquid chromatography (HPLC), utilizing an Agilent 1260 Infinity HPLC system equipped with a refractive index detector (Agilent Technologies Inc., Palo Alto, CA, United States). Analyte separation was achieved using a Bio-Rad Aminex HPX-87H column (300 × 7.8 mm, 1250140, Bio-Rad Inc, Hercules, CA, United States) and a cation-H guard column (1250129, Bio-Rad), with a flow rate of 0.5 mL/min, mobile phase of 0.02 N H_2_SO_4_, and column and detection temperatures of 50°C. The concentration of ethanol and short and medium chain fatty acids (propionic, butyric, pentanoic, hexanoic, heptanoic, and octanoic) within the filtered samples were quantified with tandem gas chromatography-mass spectroscopy (GC-MS) utilizing an Agilent 7890A GC instrument (Agilent Technologies Inc.) equipped with an L-PAL3 autosampler system (LECO Corporation, St. Joseph, MI, United States), a solid phase microextraction (SPME) fiber (StableFlex fiber assembly 23 Ga, 57298-U, MilliporeSigma, Burlington MA, United States), and a Pegasus BT TOF-MS detector (LECO Corporation). For each run, headspace sampling with the SPME fiber occurred over 20 min with agitation at 95°C followed by a sample desorb time in the injection port of 20 min. The oven temperature ramp followed the following scheme: oven equilibration of 1 min, 50°C for 2 min, ramp of 8°C per min to 250°C, then 5.5 min at 250°C for a total run time of about 30 min.

### 2.5 DNA sequencing and metagenome assembly

For metagenomic analyses, DNA was extracted from microbial biomass pellets using a published phenol-chloroform extraction procedure (Scarborough et al., 2020), but omitting the bead-beating step. DNA aliquots were submitted to the Joint Genome Institute (JGI) (Berkeley, CA, USA) for sequencing, which was performed using either paired-end 2 × 150 bp Illumina NovaSeq S4 sequencing (Illumina, Inc., San Diego, CA, USA) or single-molecule real-time (SMRT), long-read sequencing on a Sequel II platform (Pacific Biosciences, Inc. [PacBio], Menlo Park, CA, USA). DNA processing, sequencing, quality checking, assembly, binning, annotation, and taxonomy classification was performed using published procedures (Walters et al., 2022). This analysis resulted in a total of 173 metagenome-assembled genomes (MAGs), which were reported elsewhere (Walters et al., 2022).

### 2.6 Non-redundant MAG library

For microbial community analyses we used a dataset of 217 non-redundant MAGs (Supplementary Table S1), which represent the collective set of metagenomes we have assembled from several fermentation bioreactors seeded with the same inoculum but fed different carbohydrate-rich feedstocks (Myers et al., 2023). In the assembly of this non-redundant dataset we used the 173 MAGs obtained from the UFMP-fed bioreactor described in this study, 105 MAGs from a UFMP-fed upflow sludge blanket bioreactor (Walters et al., 2022), 10 MAGs from a cellulosic ethanol thin stillage bioreactor (Scarborough et al., 2018a), 8 MAGs from a xylose-rich synthetic cellulosic medium reactors (Scarborough et al., 2022b), 51 MAGs from bioreactors using starch-ethanol thin stillage as the feedstock (Fortney et al., 2022), and 48 MAGs from bioreactors fed dairy manure hydrolysate (Ingle et al., 2022). These MAGs were consolidated by dereplication (dRep, v3.2.2; “dereplicate” command with “–conW 0.5” and” –N50W 5″ custom parameters) (Olm et al., 2017), obtaining the final library of 217 non-redundant MAGs used for further analysis. This set of non-redundant MAGs has 151 MAGs assembled from Illumina and 66 MAGs assembled from PacBio sequencing and includes MAGs with greater than 75% completion and less than 7.5% contamination (Supplementary Table S1).

### 2.7 Metagenome based microbial community analysis

The presence and abundance of microorganisms in different bioreactor samples was assessed as follows (default settings used unless otherwise specified). First, sequence reads from the FASTQ sequencing files were mapped to the concatenated FASTA files of the non-redundant MAG library using Bowtie2 (v2.2.2) (Langmead and Salzberg, 2012) with the “bowtie2” command. Resulting SAM files were converted to BAM files and sorted using samtools (v1.15.1; “samtools view” and “samtools sort” commands) (Li et al., 2009). CoverM (v0.4.0) (https://github.com/wwood/CoverM) was used to generate coverage and relative abundance statistics of mapped reads using the “coverm genome” command on the sorted BAM files. For phylogenetic tree generation of MAGs and related organisms, GTDB-Tk (v1.5.1, database release 202) (Chaumeil et al., 2020) was used for alignment based on the concatenation of 120 bacterial single-copy marker genes (Bac120) (“gtdbtk identify” and “gtdbtk align” commands), RAxML-NG (v0.9.0) (Kozlov et al., 2019) for tree construction (“raxml-ng --parse” command with “--model LG+G8+F” model specification) using 1000 bootstraps, and TreeViewer (v2.0.1) for visualization.

A metabolic network containing known routes for fermentation of lactose to the seven major extracellular products observed during bioreactor operation was constructed from previously described pathways and the MetaCyc database (v.26.5) (DeMoss et al., 1951; Maxwell et al., 1962; Bissett and Anderson, 1973; Song and Lee, 2006; Pokusaeva et al., 2011; Buckel and Thauer, 2013; Cao et al., 2013; Caspi et al., 2014). This network was used as a basis for predicting gene and metabolic pathway presence within MAGs. Predictions of gene presence and metabolic pathways were done using enzyme commission numbers (EC numbers), KEGG orthology numbers (KO numbers), cluster of orthologous genes numbers (COG numbers), and gene product names described in gene annotations predicted by the JGI metagenome processing pipeline (Clum et al., 2021). The General Feature Format (gff) files containing these annotations are available on the GitHub repository https://github.com/GLBRC/UFMP-metagenomics. To supplement these annotations, the “Pathway Hole Filler” tool of the PathoLogic component of Pathway Tools (version 25.5 tier 1) (Karp et al., 2011; Karp et al., 2021) was used to search for genes predicted to be missing in reconstructed metabolic pathways. Additionally, using the Geneious Prime software (v2022.1.1), tblastn searches (default parameters; percent identity ≥25%; query coverage ≥75%) (Gerts et al., 2006) were used to identify electron confurcating lactate dehydrogenase (ecLDH) homologues by sequence comparison to the *ecLDH* gene *of Acetobacterium woodii* strain DSM 1030 (Weghoff et al., 2015) (Supplementary Table S2A). A similar tblastn approach was used to identify lactose permease homologues (Supplementary Table S2B), lactose-specific PTS permease components (Supplementary Table S2C), and lactate permease homologues (Supplementary Table S2D) (Saier et al., 2021). A summary of the predicted presence of gene homologues in the 10 most abundant MAGs is provided in Supplementary Table S3.

Phylogenetic and gene neighborhood approaches were used to infer whether identified electron transfer flavoproteins (EtfAB) were partaking in reverse β-oxidation or lactic acid utilization (Costas et al., 2017; Detman et al., 2019). First, a phylogenetic tree based on the EtfB subunit was constructed, including genes for EtfB proteins that were biochemically characterized as partaking in either reverse β-oxidation or lactic acid utilization. Inferences of the function of MAG EtfB were made for those that clustered with characterized EtfB. To corroborate these inferences, an assessment of the presence of relevant genes flanking the *etfB* genes was performed. The presence of acetyl-CoA acetotransferase (ACAT), 3-hydroxyacyl-CoA dehydrogenase (HAD), enoyl-CoA hydratase (EcoAH), and acyl-CoA dehydrogenase (ACD) genes flanking or nearby (within 5 kb) *etfAB* genes was an indication of the EtfAB pair being relevant to reverse β-oxidation, whereas presence of lactate permease (LacT) and ecLDH genes was indicative of relevance of the EtfAB pair in lactic acid utilization. For phylogenetic tree generation of EtfB amino acid sequences, MUSCLE (v3.8.31) (Edgar, 2004) was used for alignment using the “muscle” command and RAxML (v8.2.11) (Stamatakis, 2014) for tree construction (“raxmlHPC-SSE3” command with “-m PROTCATAUTO -f a” flags) using 500 bootstraps. The amino acid sequences used in this analysis are provided in Supplementary Table S4. Figure 5 was created using Biorender.com.

## 3 Results

### 3.1 Bioreactor performance

**FIGURE 1:**
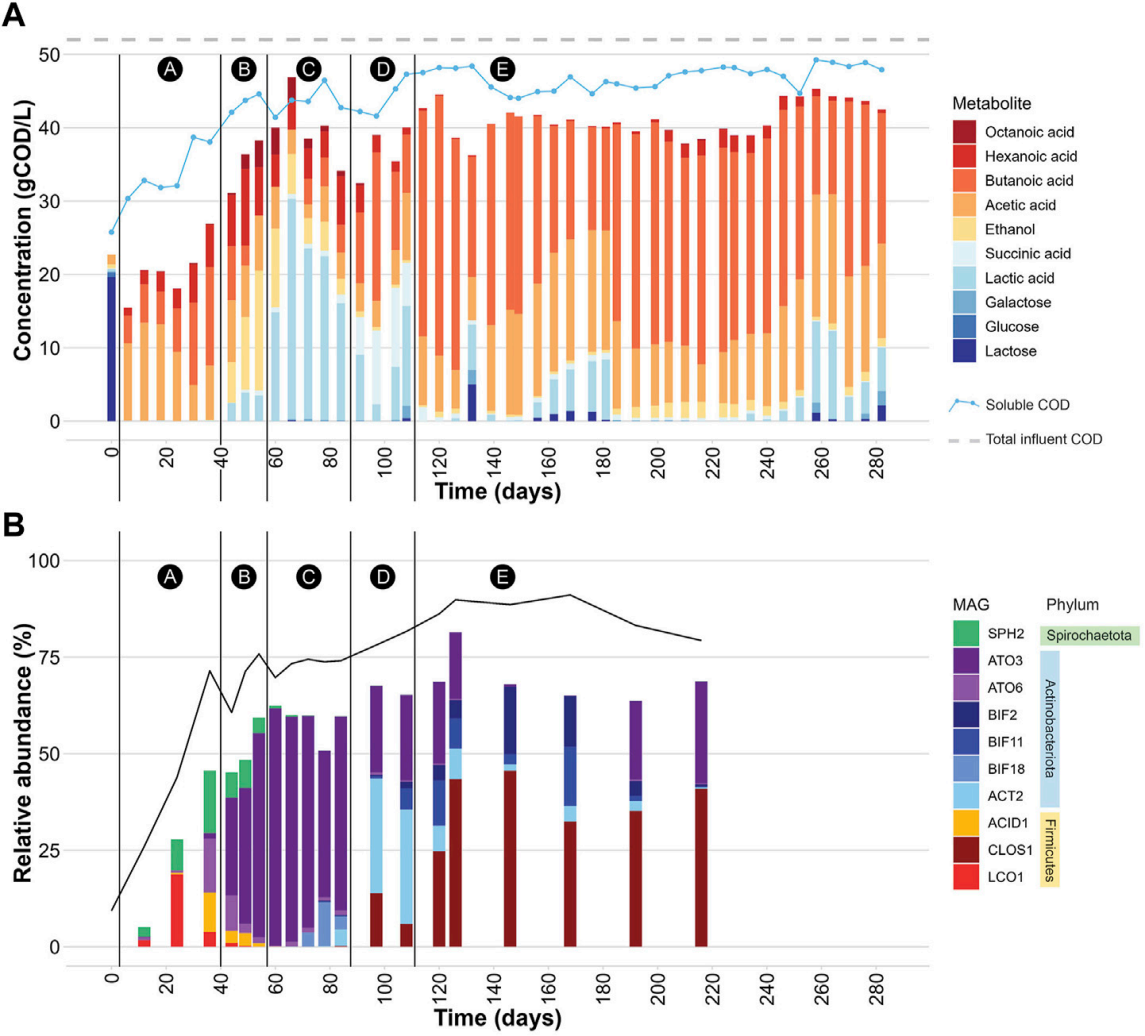
**(A)** Extracellular concentrations of carbohydrates and fermentation products during bioreactor operation. The black vertical lines demarcate 5 periods (A-E) in which different profiles of extracellular fermentation products were present. The “soluble COD” line represents the experimentally determined soluble COD concentration in gCOD/L at each time point. The “total influent COD” line represents the average total COD concentration (gCOD/L) determined for the UFMP feedstock. See also Supplementary Table S5. **(B)** Relative abundance of 10 abundant MAGs (Table 2) during bioreactor operation. The black line represents the percentage of DNA sequences that mapped to the set of 217 non-redundant MAGs. The black vertical lines demarcate periods A-E in which different profiles of extracellular fermentation products were identified. See also Supplementary Table S6.

Bioreactor performance was monitored by measuring extracellular fermentation products and carbohydrates during the operational period (Figure 1A). Lactose was metabolized and converted to mostly organic acids and ethanol (Table 1), demonstrating efficient fermentation of this sugar during the operational period. Additionally, the measured levels of phosphate and total nitrogen in the bioreactor indicated that neither of these macronutrients became limiting during bioreactor operation (Table 1). After the initial dilution of the inoculant biomass (18 days), biomass concentration showed no major sustained directional trend (Supplementary Table S5) indicating relatively consistent growth of the microbial community.

**TABLE 1:** Concentrations of nutrients and fermentation products in feedstock and bioreactor effluent.

Parameter	Feedstock^a^	Bioreactor effluent
Soluble COD (g COD/L)	51 ± 4 (n = 5)	44 ± 5 (n = 47)
Carbohydrates^b^ (g COD/L)	50 ± 5 (n = 5)	1 ± 3 (n = 47)
Organic acids^c^ (g COD/L)	1 ± 1 (n = 5)	36 ± 8 (n = 47)
Ethanol (g COD/L)	<1 (n = 5)	2 ± 3 (n = 47)
Phosphate (mg P/L)	270 ± 50 (n = 4)	310 ± 70 (n = 48)
Total nitrogen (mg N/L)	660 ± 30 (n = 4)	360 ± 80 (n = 12)

^a^ Bioreactor feedstock was UFMP supplemented with 400 mg N/L as NH_4_Cl.

^b^ Sum of lactose, glucose, and galactose. Only lactose was detected in the UFMP feedstock.

^c^ Organic acids detected include acetic, lactic, succinic, propionic, butyric, hexanoic, octanoic, formic, pentanoic, and heptanoic acid (Supplementary Table S5).

The bioreactor media initially had a high concentration of lactose because at start up half of the reactor volume was composed of the UFMP feedstock (Figure 1A). After this initial lactose was depleted, lactose and one of its monomeric sugar constituents, galactose, were only rarely detectable in the bioreactor (e.g., day 132 in Figure 1A).

The accumulation of extracellular fermentation products was dynamic during the operational period (Figure 1A), even though flow rates, temperature, and pH were kept constant. After an initial period of acclimation of the microbial community to the UFMP feedstock, about 80% of the soluble COD in the bioreactor effluent could be accounted for by the accumulation of the seven fermentation products included in Figure 1A (ethanol and lactic, succinic, acetic, butyric, hexanoic, and octanoic acids). The sum of these extracellular fermentation products represented, on average, 75% of the COD in the feedstock (Table 1). To facilitate the analysis of the bioreactor operation, we define five distinct periods chiefly based on the identity of the two most abundant fermentation products (Figure 1A); reactor performance during these periods is summarized in Supplementary Table S7:

Period A, from day 0–36, corresponds to a period when acetic, butyric, and hexanoic acids are the predominant extracellular fermentation products.

Period B, from day 44–54, is characterized by a decrease in the extracellular concentration of butyric acid and the accumulation of ethanol, acetic, lactic, hexanoic, and octanoic acids in the media.

Period C, from day 60–84, is a time in which lactic acid is the predominant extracellular product.

Period D, from day 91–108, is characterized by a decreased concentration of extracellular lactic acid and increased accumulation of butyric and succinic acids as major fermentation products.

Period E, from day 114–282, is one in which butyric and acetic acids become the major extracellular fermentation products along with the sporadic accumulation of lactic acid.

### 3.2 Microbial community characterization

The community structure throughout bioreactor operation was analyzed by mapping DNA sequence reads from 20 bioreactor samples to a set of 217 non-redundant MAGs and assessing coverage and relative abundance statistics for each MAG (Supplementary Table S6). Microbial community compositions across time points show notable differences (Figure 1B). Moreover, the operational periods A-E, which were defined based on accumulation of fermentation products (Figure 1A), differed in which community members were most abundant, showing distinct periods of microbial community structure (Figure 1B).

**FIGURE 2:**
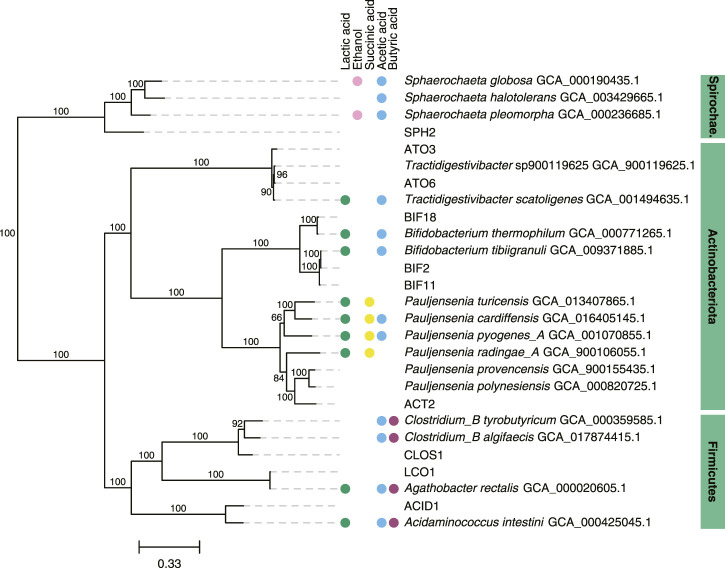
Phylogenetic tree of 10 abundant MAGs in the UFMP-fed bioreactor and related genomes. The tree was based on sequence comparisons of 120 concatenated bacterial single-copy marker genes; 1000 bootstraps were used with bootstrap values shown as a percentage. The scale bar represents evolutionary distance and indicates the number of nucleotide substitutions per sequence site. When available, metabolite production from a species reported in the literature was indicated with colored circles: green, lactic acid; magenta, ethanol; yellow, succinic acid; blue, acetic acid; purple, butyric acid (Wüst et al., 1995; Ramos et al., 1997; Hall et al., 2002; Wu and Yang, 2003; Zhu and Yang, 2004; Jumas-Bilak et al., 2007; Von Ah et al., 2007; Ritalahti et al., 2011; Dwidar et al., 2013; Wu et al., 2014; Li et al., 2015; Rosero et al., 2016; Bidzhieva et al., 2020; Eckel et al., 2020).

We focused further analysis on a set of 10 MAGs, which represented the highest relative abundances observed during the operational period of the bioreactor. We selected these 10 MAGs after sorting the maximum observed relative abundances of each MAG at all sample points (Table 2). This set of 10 MAGs includes one from the *Spirochaetota* phylum (SPH2), six from the *Actinobacteriota* phylum (ACT2, ATO3, ATO6, BIF2, BIF11, BIF18), and three from the *Firmicutes* phylum (ACID1, CLOS1, LCO1). The assembled genomes of these 10 MAGs have completeness greater than 94% and contamination less than 5%, and encompass MAGs with observed relative abundances as high as 61.5% and as low as 10.2% (Table 2). Furthermore, half of these 10 MAGs were obtained from PacBio sequencing with assembled genomes containing as few as 1 to 4 contigs, whereas the other half were obtained from Illumina sequencing and assembled into 17 to 63 contigs. Only five of these 10 high abundance MAGs had a close representative genome in the GTDB database, indicating that several of these MAGs may be derived from or represent yet uncharacterized microorganisms. To provide context, a phylogenetic analysis was conducted of these 10 MAGs compared to the most closely related isolates for which fermentation products have been reported (Figure 2).

**TABLE 2:** Abundance, taxonomy, accession numbers, genomic features, and quality statistics of the ten most abundant MAGs in the UFMP-fed bioreactor.

MAG ID	Max relative abundance (%)	Mean relative abundance (%)	Phylum^a^	Genus and species^a^	Reference genome^a^	Sequencing platform	Completeness (%)^b^	Contamination (%)^b^	MAG size (Mbp) ^b^	No. Cont-igs^b^	N_50_ (Mbp)^b^	%GC^b^	NCBI genome accession number
ATO3	61.5	25.4	*Actinobacteriota*	*Olsenella_B*	N/A	Illumina NovaSeq S4	97.98	0	2.454	28	0.1431	61.8	JALCMG000000000
CLOS1	45.6	12.1	*Firmicutes_A*	*Clostridium_B*	N/A	PacBio Sequel II	99.83	0	3.567	1	3.5672	35.0	JAKVOI000000000
ACT2	29.6	4.3	*Actinobacteriota*	*Pauljensenia*	N/A	Illumina NovaSeq S4	96.68	4.1	2.988	40	0.1187	70.5	JALCMY000000000
LCO1	18.7	1.3	*Firmicutes_A*	*Agathobacter rectalis*	GCF_000020605.1	PacBio Sequel II	99.3	0.24	3.246	4	1.6305	41.9	JAKVOQ000000000
BIF2	17.4	2.3	*Actinobacteriota*	*Bifidobacterium tibiigranuli*	GCF_009371885.1	PacBio Sequel II	99.12	4.36	3.1	3	3.0762	60.6	JAKVOD000000000
SPH2	16.1	2.3	*Spirochaetota*	RUG023	N/A	PacBio Sequel II	97.63	2.3	2.472	20	0.1891	55.9	JAKVOV000000000
BIF11	15.3	2.3	*Actinobacteriota*	*Bifidobacterium tibiigranuli*	GCF_009371885.1	Illumina NovaSeq S4	94.88	2.88	2.706	17	0.3076	60.6	JALCMO000000000
ATO6	13.9	1.7	*Actinobacteriota*	*Olsenella_B* sp900119625	GCF_900119625.1	Illumina NovaSeq S4	84.95	0	1.818	17	0.5024	62.7	JALCSG000000000
BIF18	11.4	0.9	*Actinobacteriota*	*Bifidobacterium thermophilum*	GCF_000771265.1	Illumina NovaSeq S4	87.44	0.95	1.802	63	0.0375	60.5	JALCUC000000000
ACID1	10.2	0.9	*Firmicutes_C*	*Acidaminococcus*	N/A	PacBio Sequel II	100	0.6	3.057	1	3.057	48.1	JAKVOM000000000

^a^ Taxonomy was assigned using GTDB-Tk tool and database (Chaumeil et al., 2020). Reference genome indicates the NCBI GenBank accession number of the reference genome in the GTDB that is closest to the representative MAG. Further details on MAG characterization are provided elsewhere (Walters et al., 2022). N/A refers to MAGs without a closely matched reference genome.

^b^ Quality statistics were determined with CheckM (Parks et al., 2015).

When the relative abundance of the 10 MAGs is evaluated at each sampled time point (Figure 1B), it is evident that none of these MAGs were abundant in the inoculum, suggesting that these community members were enriched at one or more periods during operation of the bioreactor. For periods B to E, there is agreement between the enriched MAGs (Figure 1B), the observed pattern of extracellular fermentation products (Figure 1A), and inferences that can be made from the phylogenetic analysis (Figure 2). These observations are further explored in conjunction with the metabolic analysis described below to infer the functional role of the abundant most abundant organisms.

### 3.3 Microbial community metabolic network analysis

**FIGURE 3:**
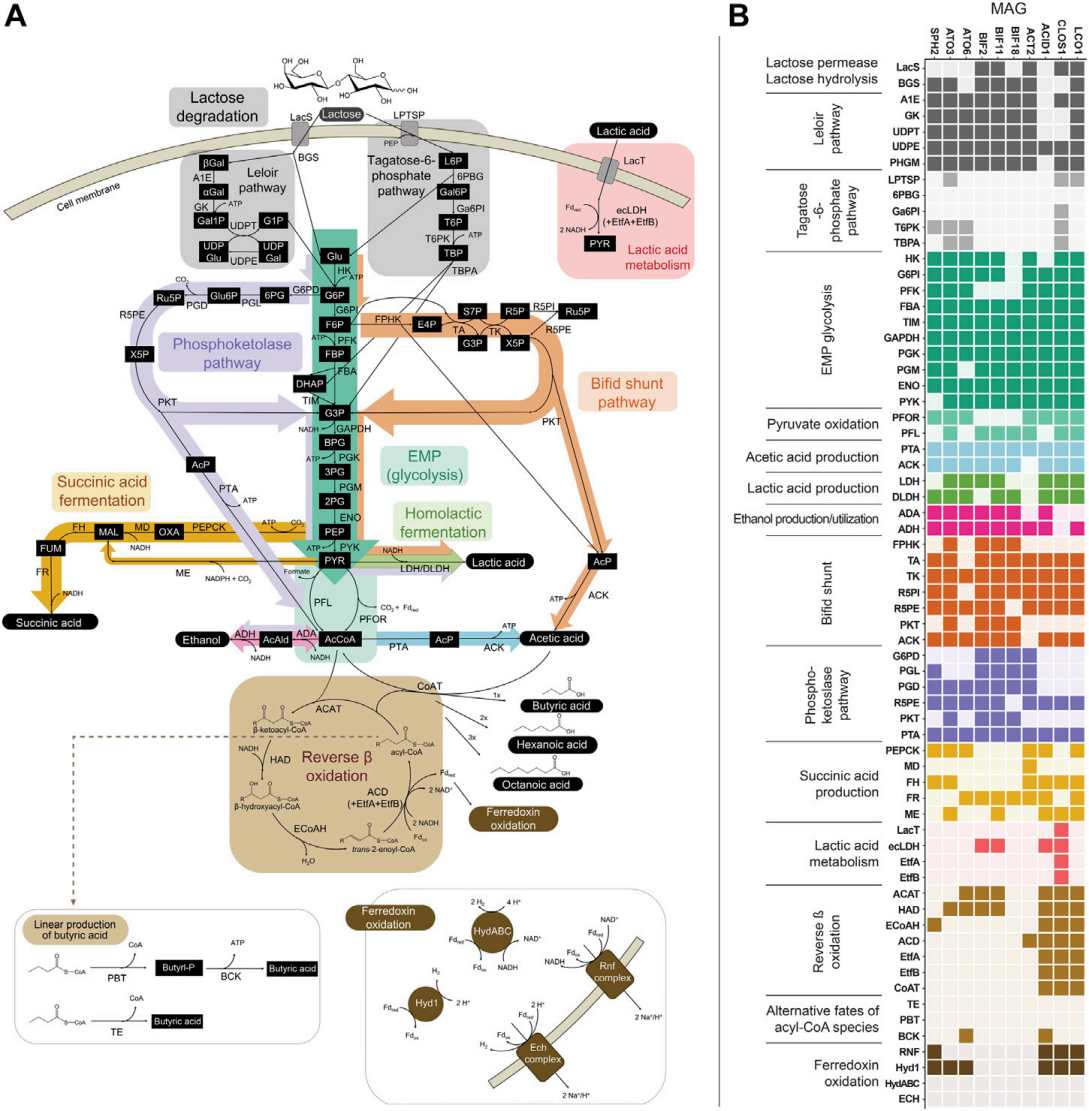
**(A)** Metabolic network of pathways predicted to be involved in the fermentation of lactose in the bioreactor. **(B)** Predicted presence of genes within the 10 abundant MAGs. Genes within Embden-Meyerhof-Parnas (EMP) glycolysis, lactic acid production, or ethanol production that are also shared with the bifid shunt and/or phosphoketolase pathway are depicted in the heatmap only once (i.e., omitted from the bifid shunt and/or phosphoketolase pathway sections), for ease of reading. Several variations for anaerobic succinic acid production exist (Song and Lee, 2006; Cao et al., 2013) and only those present within the abundant MAGs are represented here. See Supplementary Figure S1 for other succinic acid production pathways and predicted genes for each variation. Gene, pathway intermediate, and coenzyme abbreviations are provided in Supplementary Table S8.

We queried the set of 10 abundant MAGs (Table 2) for the presence of genes predicted to encode the enzymes associated with the known routes for fermentation of lactose to the seven major extracellular products observed during bioreactor operation (Figure 3). This analysis, described below, allowed us to hypothesize the role of different microbial community members in the metabolism of lactose and accumulation of the major extracellular fermentation products that were identified in the bioreactor.

#### 3.3.1 Transport and metabolism of lactose

Two routes for the transport and initial metabolism of lactose were investigated, including the Leloir-pathway-based route and the tagatose-6-phosphate pathway (Figure 3). Evidence for the presence of genes encoding both lactose permease (LacS) and β-galactosidase (BGS) was found in the *Firmicute* LCO1 and the *Actinobacteriota* ACT2, BIF11, and BIF2. In addition, genes encoding the complete Leloir pathway were found in LCO1, all the *Actinobacteriota* and the *Spirochaetota* SPH2. None of the ten MAGs were predicted to contain all the genes typically encoding proteins in the tagatose-6-phosphate pathway (Figure 3B). These data suggest that lactose degradation in the UFMP-fed bioreactor occurs primarily via the Leloir pathway and that the *Firmicute* LCO1 and the *Actinobacteriota* ACT2, BIF11, and BIF2 represent microorganisms that contribute to lactose fermentation in this microbial community.

However, none of these 10 MAGs were abundant in operational periods B and C (Figure 1B) suggesting that other organisms are responsible for lactose fermentation during those periods. While no homologue of a gene encoding LacS was identified within ATO3, it is an abundant MAG during these periods (Figure 1B), is predicted to encode proteins for BGS and the Leloir pathway, and is closely related to an organism reported capable of metabolizing lactose (*Tractidigestivibacter scatoligenes*; Figure 2) (Li et al., 2015). Combined, these observations suggest that ATO3 contributes to lactose fermentation within this microbial community despite no LacS homologue being identified within the MAG.

For the metabolism of Leloir-pathway-derived intermediates, EMP glycolysis, bifid shunt, and the phosphoketolase pathway were investigated. All of the 10 most abundant MAGs in the UFMP-fed bioreactor contained genes encoding many of the known enzymes in glycolysis. We found that three abundant *Bifidobacterium* MAGs (BIF2, BIF11, BIF18) contained genes encoding for the bifid shunt and the phosphoketolase pathway, except for a gene encoding for a homologue of R5PE within BIF18 (Figure 3B). These three abundant *Bifidobacterium* MAGs did not appear to encode for PFK, making the glycolysis pathway incomplete, suggesting their utilization of one of the heterofermentative pathways (Figure 3B), likely the bifid shunt which is characteristic of *Actinobacteriota* in the order *Bifidobacteriales* (Pokusaeva et al., 2011). A complete set of genes encoding the bifid shunt were also present in the *Actinobacteriota* ATO3, a close relative of the lactic and acetic acid producing *T. scatoligenes* (Figure 2), while the phosphoketolase pathway was incomplete (Figure 3B), suggesting organisms represented by this abundant MAG may also utilize the bifid shunt.

#### 3.3.2 Pathways for production of extracellular fermentation products

Succinic acid was a major extracellular fermentation product that accumulated during period D of bioreactor operation (Figure 1A). Out of the 10 abundant MAGs, only the *Actinobacteriota* ACT2 encodes the genes necessary for succinic acid production via the route utilizing phosphoenolpyruvate carboxykinase (PEPCK) and malate dehydrogenase (MD), while only the *Firmicutes* ACID1 and LCO1 encode the genes necessary for succinic acid production via the route through pyruvic acid utilizing malic enzyme (ME) (Figure 3B, Supplementary Figure S1). Complete sets of genes for the other known routes of succinic acid production were not detected in the 10 abundant MAGs (Supplementary Figure S1). Of the abundant MAGs predicted to encode enzymes in succinic acid production pathways, only ACT2 was abundant concomitantly with the accumulation of extracellular succinic acid (Figure 1). ACT2 was also found to contain all the genes for lactose utilization via the Leloir pathway, a complete set of genes encoding the glycolysis pathway, and incomplete phosphoketolase and bifid shunts (Figure 3B). Additionally, ACT2 phylogenetically clustered near organisms shown to produce succinic acid (Figure 2). Combined, these data suggest that microorganisms represented by the ACT2 MAG were significant contributors to extracellular succinic acid accumulation in the bioreactor, likely from direct utilization of lactose via a combination or the Leloir and glycolytic pathways.

Ethanol was a major fermentation product that accumulated during period B of reactor operation and, to a lesser extent, periods C and E (Figure 1A). The SPH2, ATO3, BIF2, and BIF11 MAGs contained genes encoding complete or nearly complete routes for lactose metabolism, glycolysis or the phosphoketolase pathway, and ethanol production (Figure 3B), suggesting they may have contributed to ethanol production from lactose within the microbial community either through heterofermentation via the phosphoketolase pathway or glycolysis-based fermentation. Of these MAGs, only SPH2 was both highly abundant during period B (Figure 1B), and phylogenetically related to organisms shown to produce ethanol (Figure 2), suggesting it may have been a significant contributor to ethanol production during period B.

Butyric, hexanoic, and octanoic acids, organic acids often derived from the reverse β-oxidation pathway, accumulated at different times during bioreactor operation, with butyric acid being one of the main extracellular products along with acetic acid during period E (Figure 1A). The three abundant *Firmicute* MAGs (ACID1, CLOS1, LCO1) contained all the genes encoding enzymes in the reverse β-oxidation pathway (Figure 3B) and are related to known chain elongators (Figure 2), suggesting that members of the *Firmicutes* represented by these three MAGs are contributors to the extracellular accumulation of butyric, hexanoic, and octanoic acids in this microbial community. Additionally, alternative routes through which butyric acid can be produced involve either thioesterase (TE) or the combination of phosphate butyryltransferase (PBT) and butyrate kinase (BCK) as the terminal enzymes acting upon butyryl-CoA. We only detected a BCK homologue in ACID1, but no gene homologues for TE or PBT in the *Firmicute* MAGs (Figure 3B), suggesting that butyric acid was not produced by these alternative routes.

**FIGURE 4:**
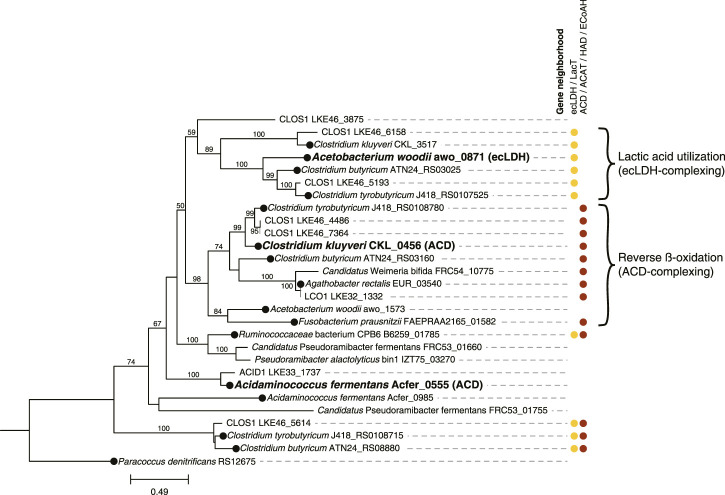
Phylogenetic tree comparing electron transfer flavoprotein B (EtfB) amino acid sequences of ACID1, CLOS1, and LCO1 with other EtfB sequences. 500 bootstraps were used with bootstrap values shown as a percentage. Bootstrap values below 50 are excluded from the figure. The scale bar represents evolutionary distance and indicates the number of amino acid substitutions per sequence site. Locus numbers for *etfB* genes are provided. Three EtfB proteins with biochemically characterized enzymatic function were used as references and indicated with bold text; the protein with which they complex is indicated in parentheses (ACD, acyl-CoA dehydrogenase; ecLDH, electron confurcating lactate dehydrogenase) (Li et al., 2008; Chowdhury et al., 2014; Weghoff et al., 2015). The predicted protein sequence of *Paracoccus denitrificans* RS12675 was used as an outgroup *etfb* from a non-*Firmicute* isolate with no biochemical evidence for electron bifurcation (Roberts et al., 1999; Costas et al., 2017). EtfB sequences derived from isolated organisms are indicated with a black circle at the node. Gene neighborhood designations describe the presence of one or more relevant genes flanking or nearby the *etfB* genes with ecLDH/LacT (electron confurcating lactate dehydrogenase/lactate permease) relevant to lactic acid utilization and ACD/ACAT/HAD/ECoAH (acyl-CoA dehydrogenase/acetyl-CoA acetotransferase/3-hydroxyacyl-CoA dehydrogenase/enoyl-CoA hydratase) relevant to reverse β-oxidation. Brackets indicate proposed clusters of EtfBs that participate in either lactic acid utilization or reverse β-oxidation. The raw sequences used to generate this tree are available in Supplementary Table S4.

Because reverse β-oxidation can use intermediates originating from the metabolism of several electron donating compounds including carbohydrates, lactic acid, or ethanol, we investigated the presence of gene homologues necessary for the utilization of each of these three electron donors. As part of this analysis, we performed a phylogenetic comparison and gene neighborhood analysis of the EtfBs identified in the three *Firmicute* MAGs and including biochemically characterized EtfBs for reference (Figure 4).

Out of the three *Firmicute* MAGs only LCO1 contained the genes needed for lactose utilization, and in addition, there was no evidence for the presence of the LacT, ecLDH, nor EtfAB genes needed for lactic acid utilization, or the ADA gene needed for ethanol utilization (Figure 3B; Figure 4), suggesting that LCO1 performs chain elongation with lactose as the organic substrate. This inference is in agreement with properties of the closely related *Agathobacter rectalis* (Figure 2), an organism reported to be capable of reverse β-oxidation and lactose fermentation, while incapable of fermenting lactic acid (Duncan et al., 2004; Rosero et al., 2016).

The CLOS1 MAG contained the genes encoding enzymes for lactic acid utilization and did not appear to have a complete set of genes for lactose metabolism or ethanol utilization (Figure 3B). The CLOS1 MAG contained multiple pairs of EtfAB homologues, whereas the LCO1 and ACID1 MAGs only had one EtfAB pair (Figure 4). Two of the EtfB homologues in CLOS1 (LKE46_5193 and LKE46_6158 in Figure 4) clustered with an EtfB of *A. woodii* (awo_0871 in Figure 4) that has been shown to facilitate lactic acid utilization in complex with ecLDH (Weghoff et al., 2015). In addition, all EtfBs in this same cluster were found to be located adjacent to genes that encode lactic acid utilization proteins (e.g., LacT and ecLDH) and many originated from known lactic acid utilizing organisms (Figure 4) (Li et al., 2008; Dwidar et al., 2013; Zhu et al., 2017; Detman et al., 2019). Thus, this analysis supports the inference that CLOS1 uses lactic acid as a substrate for chain elongation. Moreover, CLOS1 was abundant only when butyric acid was the main elongation product, suggesting that chain elongation in CLOS1 uses lactic acid as the substrate and produces butyric acid as an end product.

The ACID1 MAG contained a gene encoding ecLDH but genes encoding LacT and the EtfAB associated with lactic acid utilization were not found (Figure 3B; Figure 4), suggesting ACID1 did not use lactic acid as the substrate for chain elongation. Furthermore, genes encoding lactose utilization were not found (Figure 3B), suggesting ACID1 was not using lactose as a substrate. Notably, the ACID1 MAG encoded genes of enzymes needed for ethanol production or utilization (Figure 3B) (Seedorf et al., 2008). While it remains unclear, this analysis suggests that ACID1 possibly utilized ethanol as an electron donor for chain elongation.

## 4 Discussion

**FIGURE 5:**
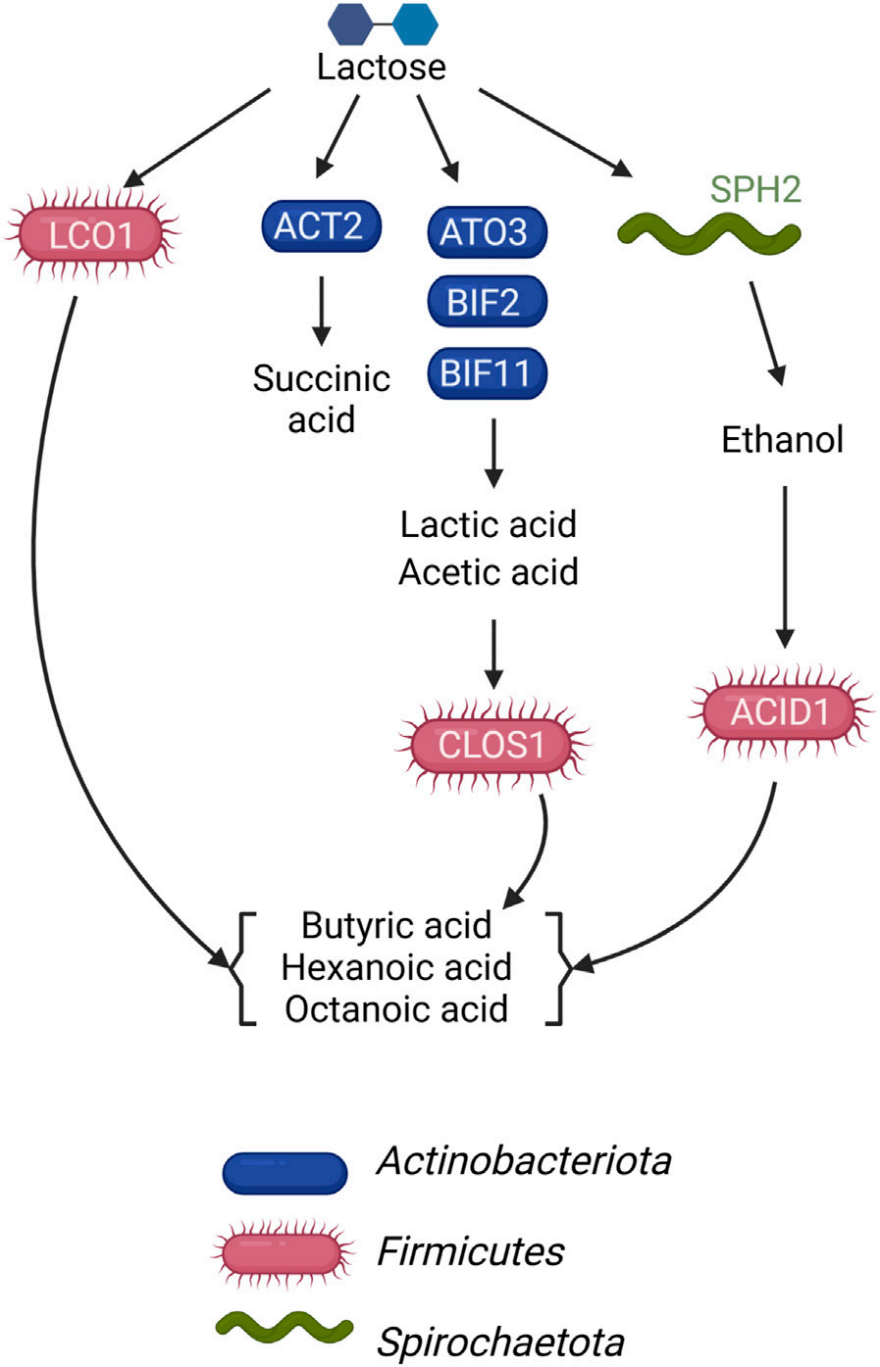
Model of metabolic networks in the microbial community established in the UFMP-fed bioreactor. Arrows indicate the direction of carbon flow from the lactose of the feedstock and through intermediates as they are transformed by microbial community members.

This study sought to determine if the metagenomic features of a microbial community fermenting a lactose-rich residue could be used to hypothesize the function of specific members of the community in fermentation process. We were able to construct a conceptual microbial community structure model containing hypothesized roles and interactions in which *Actinobacteriota* ferment lactose via the Leloir and bifid shunts, with lactic and acetic acids as major fermentation products, and *Firmicutes* contribute to the production of butyric, hexanoic, and octanoic acids by chain elongation, but using different organic carbon sources as substrates (Figure 5). We also propose the functions of a succinic acid-producing *Actinobacteriota* and an ethanol-producing *Spirochaetota* in this microbial community (Figure 5). The metagenomic data indicated that some *Firmicutes* in the microbial community have the potential to completely metabolize lactose, while others do not appear to have a complete set of genes for lactose utilization and are likely using lactic acid, ethanol, or another compound as their electron donating substrate. Follow-up metatranscriptomic analyses could be effective in investigating the hypothesized roles and interactions of these community members (Scarborough et al., 2018a).

We propose that the following features are emerging from this and other studies (Costas et al., 2017; Scarborough et al., 2018a; Detman et al., 2019; Scarborough et al., 2020; Crognale et al., 2021; Sheridan et al., 2022) as cornerstone elements that will help infer the role of key members in a microbial community fermenting carbohydrate-rich feedstocks and producing one or multiple organic acids as the main products of chain elongation.

### 4.1 Microorganisms fermenting carbohydrates to lactic acid depend on feedstock composition

An emerging picture from analyzing lactic acid-producing communities at the metagenomic and metatranscriptomic level (Scarborough et al., 2018a; Crognale et al., 2021; Myers et al., 2023) is that the microorganisms enriched will likely depend on the composition of the feedstock. For instance, lactic acid producing bacteria in the UFMP-fed bioreactor described here were characterized by having the Leloir pathway for lactose metabolism and were mostly *Actinobacteriota* with a complete bifid shunt that suggested a heterofermentative process with lactic and acetic acids as the main products. In another lactose-fermenting microbial community, *Actinobacteriota* were also the most abundant community members inferred to produce lactic acid (Duber et al., 2018). In contrast, the lactic acid producing bacteria enriched in a xylose-rich lignocellulosic feedstock were primarily *Firmicutes* in the *Lactobacillaceae* family and encoded pathways for pentose utilization (Scarborough et al., 2018a). Likewise, members of the *Lactobacillaceae* family were enriched in a bioreactor fed thin stillage derived from a starch ethanol production (Fortney et al., 2021; Fortney et al., 2022). Within microbial communities fed food waste extract, the dominant lactic acid producing bacteria differed based on the organic loading rate, with *Firmicutes* in the *Streptococcaceae* family being dominant at low loading rates and *Actinobacteriota* within the *Atopobiaceae* family being dominant at high loading rates (Crognale et al., 2021). The implications of these observations are twofold. First, it suggests that a community producing lactic acid from one feedstock may not have the ability to efficiently produce lactic acid when fed another feedstock having a markedly different composition. On the other hand, our observations also suggest that a feedstock that contains a mixture of carbohydrates would enrich for microorganisms capable of fermenting one or more of those carbohydrates. Predicting which microorganisms could be enriched on a particular feedstock will require knowledge of their genetic potential to metabolize the specific carbohydrates in the feedstock.

### 4.2 Association between *Actinobacteriota* and *Firmicutes*

The inferred connection between some *Actinobacteriota* fermenting lactose to lactic acid and some *Firmicutes* utilizing lactic acid for chain elongation derived from this study has been described or hypothesized by others for microbial communities fermenting other carbohydrate-rich feedstocks (Scarborough et al., 2018a; Duber et al., 2018; Carvajal-Arroyo et al., 2019; Crognale et al., 2021; Liu et al., 2022), including within human gut microbial communities (Duncan et al., 2004; Belenguer et al., 2006). For instance, Scarborough et al. analyzed the microbial community in a bioreactor producing acetic, butyric, hexanoic, and octanoic acids from a xylose-rich feedstock and proposed that *Actinobacteriota* of the *Coriobacteriaceae* family fermented xylose to lactic and acetic acid, while members of the *Eubacteriaceae* family of *Firmicutes* produced hexanoic and octanoic acid from chain elongation of lactic acid (Scarborough et al., 2018a). A similar association was proposed by Carvajal-Arroyo et al. for a granular bioreactor fermenting thin stillage, where *Actinobacteriota* members of the *Coriobacteriaceae* family were proposed to ferment the sugars in this feedstock to lactic acid and *Firmicutes* in the *Ruminococcaceae* family were proposed to elongate lactic acid to hexanoic acid (Carvajal-Arroyo et al., 2019). In a bioreactor converting food waste extract into hexanoic acid, Crognale et al. proposed that *Pseudoramibacter alactolyticus* utilized lactic acid for chain elongation, with the lactic acid produced by *Actinobacteriota* (Crognale et al., 2021). Furthermore, Belenguer et al. used human gut strains in an isotope tracer experiment to demonstrate the flow of carbon from *Bifidobacterium adolescentis* (*Actinobacteriota*) to butyrate-producing *Firmicutes* (Belenguer et al., 2006).

### 4.3 Inferring the substrate used for chain elongation

There were 3 *Firmicutes* among the 10 most abundant MAGs in the UFMP-fed bioreactor analyzed in this study. Based on the genomic information for each MAG (Figure 3B), their abundance (Figure 1B), and the bioreactor fermentation product profiles (Figure 1A), we proposed that all three *Firmicutes* performed chain elongation, but from different substrates. CLOS1 was hypothesized to use lactic acid, LCO1 was hypothesized to use lactose, and the substrate utilized by ACID1 for chain elongation was unclear but hypothesized to be something other than lactose or lactic acid and possibly ethanol (Figure 5). The following genomic features emerged as the key features that allowed us to make these inferences.

#### 4.3.1 Chain-elongating *Firmicutes* that utilize lactic acid

To infer whether a chain elongating *Firmicute* can utilize lactic acid we propose that is necessary to identify the presence of genes that encode LacT, ecLDH, and the electron transfer flavoproteins EtfAB that are specifically associated with ecLDH. In our community, the CLOS1 MAG was the only MAG among the 10 most abundant that contained all these genes (Figure 3B). The presence of genes encoding LacT, ecLDH, and EtfAB were also genomic features used to identify the lactic acid-utilizing *Candidatus* Pseudoramibacter fermentans in an MCFA-producing microbial community receiving a cellulosic residue as the feedstock (Scarborough et al., 2020). Similarly, Crognale et al. used the presence of these same genes to infer the lactic acid utilizing capabilities of a *P. alactolyticus* MAG in a microbial community receiving food waste extract as the feedstock (Crognale et al., 2021). Additionally, a recent study used transcriptomics to show that these genes were upregulated within the gut isolate *Anaerobutyricum soehngenii* when using lactic acid as a substrate for butyric acid production compared to when using glucose as a substrate (Sheridan et al., 2022).

However, since acyl-CoA dehydrogenase (ACD) in the reverse β-oxidation pathway also uses EtfAB, all chain elongating *Firmicutes* are expected to encode EtfAB (Li et al., 2008; Weghoff et al., 2015; Costas et al., 2017). Thus, for an accurate evaluation of lactic acid utilization we see a need to evaluate whether the encoded EtfAB enzyme can be predicted to form a complex with ecLDH for the oxidation of lactic acid to pyruvate. This is particularly important if there are several genes encoding EtfAB pairs identified in a genome. A recent analysis of the electron-transferring flavoprotein family provides an approach to do this, since amino acid sequences of EtfAB pairs associated with lactic acid utilization were differentiable from EtfAB pairs used by ACD (Costas et al., 2017). These phylogenetic distinctions may be able to be corroborated by gene neighborhood considerations, as genes relevant to a given function (e.g., lactic acid utilization or reverse β-oxidation) often flank *etfAB* genes (Costas et al., 2017; Detman et al., 2019). Based upon this rationale, we used phylogenetic and corroborating gene neighborhood analyses to support the inference that CLOS1 uses lactic acid as a substrate for chain elongation and rule out lactic acid utilization by the two other *Firmicutes* (Figure 4). Notably, this same analysis aided in the identification of MAGs that were capable of chain elongation.

To further evaluate the use of LacT along with ecLDH and its corresponding EtfAB pair as genomic features to infer chain elongation from lactic acid, we analyzed the genomes of three isolates capable of using lactic acid as a substrate for chain elongation (*Ruminococcaceae* bacterium CPB6, *Clostridium butyricum* KNU-L09, and *Clostridium tyrobutyricum* DSM 2637 /ATCC 25755) (Li et al., 2008; Dwidar et al., 2013; Zhu et al., 2017; Detman et al., 2019) (Supplementary Table S9). For context, organisms shown to be capable of reverse β-oxidation but not utilize lactic acid (*Clostridium kluyveri* DSM 555, *Fusobacterium prausnitzi* A2-165, and *Agathobacter rectalis* DSM 17629) (Bornstein and Barker, 1948; Duncan et al., 2004) were included in the analysis. All of the lactic acid utilizing organisms encoded LacT and ecLDH (Supplementary Table S9). Furthermore, 2 of the 3 lactic acid utilizing isolates (all except *R.* CPB6) encoded an EtfB that was both flanked by lactic acid utilizing genes and clustered with an EtfB biochemically shown to complex with ecLDH (Figure 4), in agreement with the proposed use of these genomic features.

The genome of *R.* CPB6 encoded only one EtfAB pair, which was flanked by both an ACD and a LacT. The EtfB of *R*. CPB6 did not cluster with the biochemically characterized EtfB of *C. kluyveri* nor that of *A. woodii* (Figure 4) but was in a separate cluster with the EtfB of *Ca*. P. fermentans and *P. alactolyticus*, both proposed to use lactic acid for chain elongation (Scarborough et al., 2020; Crognale et al., 2021). The flanking of genes associated with both lactic acid utilization and reverse β-oxidation suggest the possibility that this phylogenetically different EtfB could be involved in both functions, as hypothesized before for *Ca*. P. fermentans (Scarborough et al., 2020). Overall, these results are consistent with LacT, ecLDH, and EtfAB pairs associated with lactic acid utilization being appropriate genomic features to infer *Firmicutes* capable of using lactic acid for chain elongation.

Many challenges arise in inferring functions of community members in metagenome-based studies, including metabolic plasticity involving lactic acid production or consumption by chain elongators. For example, an organism may switch between lactic acid production and chain elongation as primary modes of metabolism depending on the environmental conditions (Esquivel-Elizondo et al., 2021). As another example, an organism may primarily utilize chain elongation when provided one substrate (e.g., lactic acid), but not when provided another (e.g., glucose), despite exhibiting growth on both substrates (Weimer and Moen, 2013). We propose that the analysis described here addresses the challenge presented in the first situation, whereby the identification of gene homologues within a MAG encoding LacT, ecLDH, (distinct from LDH), and an EtfAB pair that phylogenetically and through gene neighborhood analyses are predicted to be active in the lactic acid utilization pathway, help distinguish an organism as being capable of lactic acid utilization as opposed to solely lactic acid production. Metatranscriptomic data would further aid in addressing this challenge by showing the relative expression of these genes. The challenge presented in the second situation could be addressed using metatranscriptomic data, in conjunction with the analyses described here, whereby knowledge of the relative expression of each pathway could help determine which pathways are active under each condition.

#### 4.3.2 Chain-elongating *Firmicutes* that directly utilize carbohydrates as substrates

The genomic data allowed us to infer that the LCO1 MAG was the only abundant *Firmicute* MAG having complete pathways for metabolism of lactose (Figure 3B). In addition, we did not find in the LCO1 MAG genes encoding ADA, LacT, ecLDH, nor an EtfAB predicted to complex with ecLDH and facilitate lactic acid oxidation (Figure 4). Thus, we propose that the presence of genes required for carbohydrate utilization and the presence of genes required for chain elongation are a combination of genomic features that can be used to infer an organism is capable chain elongation using a carbohydrate as a substrate. In this particular case, the absence of genes required for lactic acid and ethanol utilization aided in inferring that organisms represented by LCO1 were utilizing lactose for chain elongation. The work of Scarborough et al. in the description of *Ca*. P. fermentans and *Candidatus*. Weimeria bifida (Scarborough et al., 2020) also supports this proposal since the xylose-utilizing *Ca*. W. bifida was shown to have genes encoding for the pentose phosphate pathway for xylose utilization but lacked genes encoding LacT and ecLDH. In addition, *Ca.* W. bifida only had one EtfAB pair and, in the EtfB tree (Figure 4), it belonged to the cluster predicted to be associated with reverse β-oxidation along with the biochemically characterized EtfB of *C. kluyveri*. To test the efficacy of this approach to infer chain elongation from carbohydrates, we also analyzed the genomes of *Agathobacter rectalis* ATCC 33656 and *Faecalibacterium prausnitzii* A2-165, which are isolated chain-elongating *Firmicutes* known to be incapable of utilizing lactic acid as a substrate (Duncan et al., 2004), and found that, as described for LCO1 and *Ca*. W. bifida, the genomes of these isolates did not contain genes encoding LacT, ecLDH, nor an EtfAB pair predicted to complex with ecLDH (Figure 4 and Supplementary Table S9).

#### 4.3.3 Chain-elongating *Firmicutes* that utilize ethanol

Our attempt to infer a chain elongation substrate utilized by the ACID1 was less than conclusive. It is possible that organisms represented by this MAG could utilize ethanol as a substrate for chain elongation based on the genomic observations that ACID1 is the only *Firmicute* MAG predicted to encode acetaldehyde dehydrogenase (ADA) and alcohol dehydrogenase (ADH), and the absence of genes encoding proteins needed for lactose utilization, which ruled out chain elongation from carbohydrates, and the absence of LacT and an EtfAB pair that could be associated with ecLDH, which ruled out chain elongation from lactic acid (Figure 3B). It is also possible that other substrates could be the primary substrate for ACID1. While ADA and ADH have been described as enzymes used for the conversion of ethanol to the acetyl-CoA that is needed for chain elongation (Seedorf et al., 2008), they also facilitate ethanol production and are common to many non-chain elongating taxa, leading to some ambiguity. Indeed, of the 10 most abundant MAGs analyzed from the UFMP-fed bioreactor, seven MAGs were predicted to encode one or more ADA and ADH (Figure 3B).

In the future it would be ideal to identify other genomic features that could be associated with chain elongation from ethanol. Chain elongation with ethanol as a substrate has been studied mostly in *C. kluyveri*, where the genes encoding ADA and ADH are located near genes encoding microcompartment proteins and ADA and ADH are hypothesized to microcompartmentalize (Hillmer and Gottschalk, 1972; Seedorf et al., 2008). If further studies reveal that the microcompartmentalization of ADA and ADH proves characteristic of ethanol-consuming chain elongators, related genomic features such as the presence of microcompartment genes flanking ADA and ADH may prove useful in identifying MAGs capable of chain elongation from ethanol. Notably, homologues of the *C. kluyveri* microcompartment genes (CKL_1072 and CKL_1073) were not identified within ACID1.

### 4.4 Where does chain elongation stop?

Although we have proposed genomic features that could be diagnostic for whether the substrate for chain elongation is lactic acid, ethanol, or a carbohydrate, we were not able to propose a genomic feature diagnostic of the length of the terminal product of chain elongation. Our proposal of butyric acid as the major chain elongation product of CLOS1 (Figure 5) was based on the observation that this MAG was abundant only when butyric acid was the main product of chain elongation. Our inferences regarding LCO1 and ACID1 producing longer chain fatty acids were made using similar criteria. Isolated organisms have shown a preference for chain elongation products of different lengths, (e.g., *C. tyrobutyricum* produces butyric acid while CPB6 produces butyric and hexanoic acids) (Wu and Yang, 2003; Zhu et al., 2017) and recent studies utilizing cell-free prototyping to screen enzyme homologs have indicated that thioloases (ACAT) and terminal enzymes of reverse β-oxidation are key determinants in chain length selection (Tarasava et al., 2022; Vögeli et al., 2022). These findings warrant further investigation regarding their implementation in elucidating genomic features for the determination of chain length specificity, possibly through a phylogenetic comparison of enzyme homologs. Furthermore, factors independent of genomic considerations have been implicated in the selection of chain length through thermodynamic-based effects, (e.g., hydrogen partial pressure and substrate type and concentration), exemplifying the need to consider these parameters when designing MCFA-producing bioreactors targeting specific chain lengths (Cavalcante et al., 2017; Scarborough et al., 2018a).

### 4.5 Concluding remarks

The ability to use microbial communities to generate valuable fermentation products from organic feedstocks will ultimately depend on having the knowledge to control the members of the microbial community and directing it to produce the product of interest while minimizing the accumulation of other fermentation products (Lawson et al., 2019). Our analysis adds to the growing number of approaches for analyzing, predicting, and eventually controlling fermenting microbial communities (Han et al., 2019). The following are genomic features that we propose as diagnostic features to identify the roles of key members of these microbial communities: 1) metabolic pathways for carbohydrate utilization, 2) the reverse β-oxidation pathway, including the ACD-complexing electron transfer flavoproteins (EtfAB), 3) the presence of LacT, ecLDH, and ecLDH-complexing EtfAB pair, 4) presence of ADA and ADH, and 5) predicted absence of genomic features that identify chain elongation from specific substrates. Notably, complications remain in evaluating the absence of a genomic feature due to either incomplete assembly of metagenomes or a missed gene prediction. As such, inferences should be made with caution, ideally with corroborating information such as phylogenetic inferences, correlation between MAG abundance and product accumulation, or metatranscriptomic data. Fortunately, as long-read sequencing becomes more prevalent and as gene prediction tools improve, this limitation will decrease in its practical impact. Finally, genomic features that could be used for predicting the terminal acid in chain elongation (e.g., butyric, hexanoic, or octanoic acid) or improving prediction of chain-elongation from ethanol remain knowledge gaps that require further investigation.

## Data Availability

The datasets presented in this study can be found in online repositories. The names of the repository/repositories and accession number(s) can be found below: https://www.ncbi.nlm.nih.gov/, PRJNA768492. Direct links to each metagenome are provided in Supplementary Table S6. Direct links to the MAGs are provided in Supplementary Table S1.
