# Supplementary material for: A metagenome-level analysis of a microbial community fermenting ultra-filtered milk permeate
Source: Front Bioeng Biotechnol. 2023 May 17;11:1173656. doi: 10.3389/fbioe.2023.1173656 (PMC10263058; doi:10.3389/fbioe.2023.1173656)
Supplement: Supplementary file 1 [file DataSheet1.PDF]

Supplemental figure

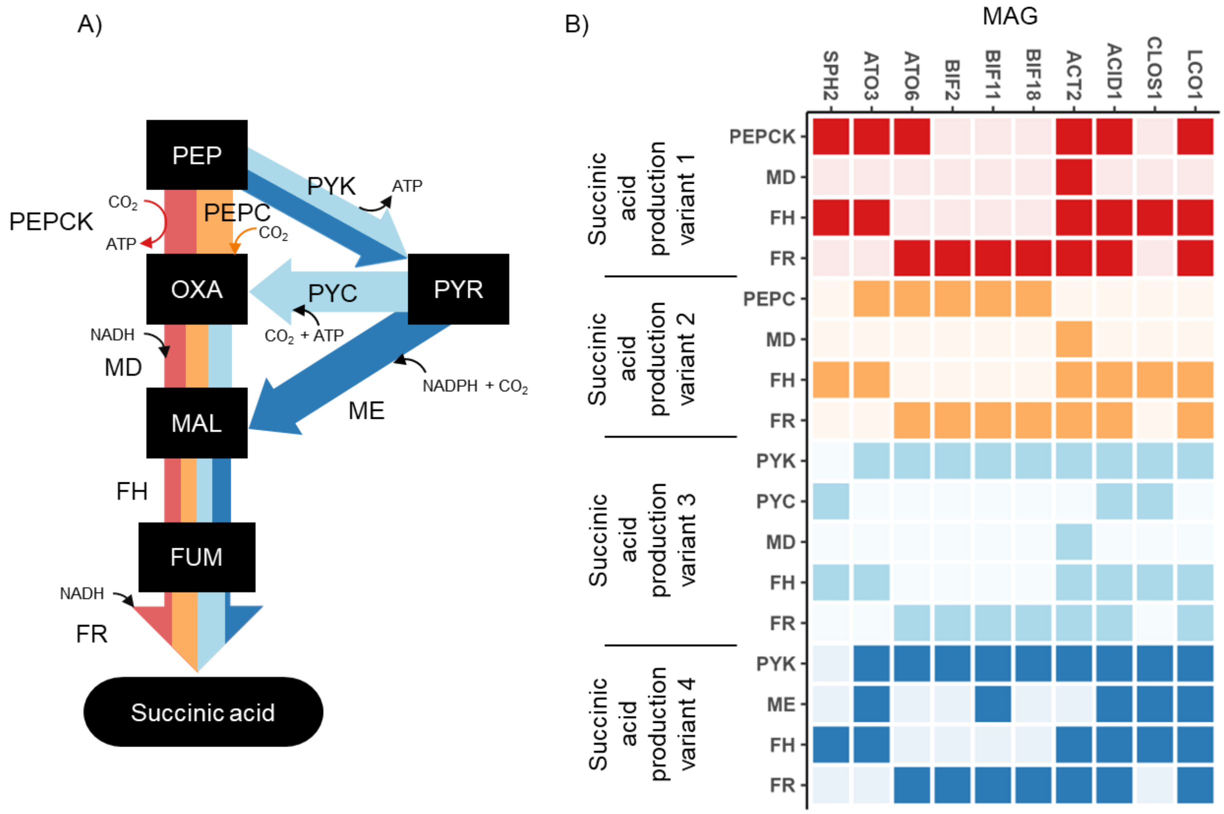

Figure S1. (A) The four anaerobic pathways for the production of succinic acid. **(B)** Predicted presence of genes within the 10 abundant MAGs. Variants 1 and 4 were present within the abundant MAGs and so are represented in Figure 5. Gene abbreviations are as follows: PEPCK, phosphoenolpyruvate carboxykinase (EC:4.1.1.32, EC:4.1.1.49, K01596, K01610); MD, malate dehydrogenase (EC:1.1.1.37, EC:1.1.5.4, K00025, K00116); FH, fumarate hydratase/fumarase (EC:4.2.1.2, K01676); FR, fumarate reductase (EC:1.3.5.4, K00244); PEPC, phosphenolpyruvate carboxylase (EC:4.1.1.31); PYK, pyruvate kinase (EC:2.7.1.40, K00873); PYC, pyruvate carboxylase (EC:6.4.1.1); ME, malic enzyme (EC:1.1.1.38). Pathway intermediate and coenzyme abbreviations are as follows: PEP, phosphoenolpyruvic acid; PYR, pyruvic acid; OXA, oxaloacetate; MAL, malate; FUM, fumarate; ATP, adenosine triphosphate; NADH, reduced nicotinamide adenine dinucleotide; NADPH, reduced nicotinamide adenine dinucleotide phosphate.
